# Elevated IgE Levels—An Allergy or an Underlying Inborn Error of Immunity in Children with Recurrent Infections?

**DOI:** 10.3390/antib12040070

**Published:** 2023-11-03

**Authors:** Polina Kostova, Vera Papochieva, Dimitrinka Miteva, Bilyana Georgieva, Sirma Mileva, Martin Shahid, Tsvetelin Lukanov, Guergana Petrova

**Affiliations:** 1 Pediatric Department, Medical University Sofia, 2 Zdrave Str., 1431 Sofia, Bulgaria; 2 Pediatric Clinic, UMHAT Alexandrovska, 1 Georgi Sofiyski Str., 1431 Sofia, Bulgaria; 3 Department of Dermatology and Venereology, Medical University Sofia, 2 Zdrave Str., 1431 Sofia, Bulgaria; 4 Dermatology Clinic UMHAT Alexandrovska, 1 Georgi Sofiyski Str., 1431 Sofia, Bulgaria; 5 Department of Clinical Immunology with Stem Cell Bank, Medical University Sofia, 2 Zdrave Str., 1431 Sofia, Bulgaria; 6 Clinic of Immunology, UMHAT Alexandrovska, 1 Georgi Sofiyski Str., 1431 Sofia, Bulgaria

**Keywords:** immunoglobulin E, allergic diseases, inborn errors of immunity

## Abstract

Elevated immunoglobulin E (IgE) is a hallmark of allergic diseases. However, high IgE levels also occur in a number of other infectious and noninfectious diseases. In most cases, elevated IgE levels indicate allergy, eczema, or chronic skin infection. Very high IgE levels are not uncommon in patients with active eczema but more often indicate monogenic atopic disorder or inborn errors of immunity with an atopic phenotype. We conducted a retrospective study of 385 children with suspected immune deficiency referred to the clinic over a 9-year period. Measurement of IgE, IgG, IgA, IgM, and IgG subclasses in blood samples revealed that nearly one-third of the patients had elevated serum IgE levels. Most of the cases with elevated IgE were children with underlying atopy—mainly atopic dermatitis and, to a lesser extent, bronchial asthma—whereas 40.12% (37 children) had no atopy at all. In the most severe cases (with extremely elevated IgE or severe dermatitis), we confirmed genetic mutations for underlying immunodeficiency. Our results indicate that allergic phenotype should not be underestimated and that children with more severe allergic disease should be evaluated for an underlying inborn error of immunity. If inborn error of immunity (IEI) is suspected, a comprehensive immunologic evaluation is required. Genetic testing helps identify the specific genetic abnormality, which provides important insight into the immunopathogenesis of the disease and accurate determination of optimal therapy.

## 1. Introduction

**Figure 1:**
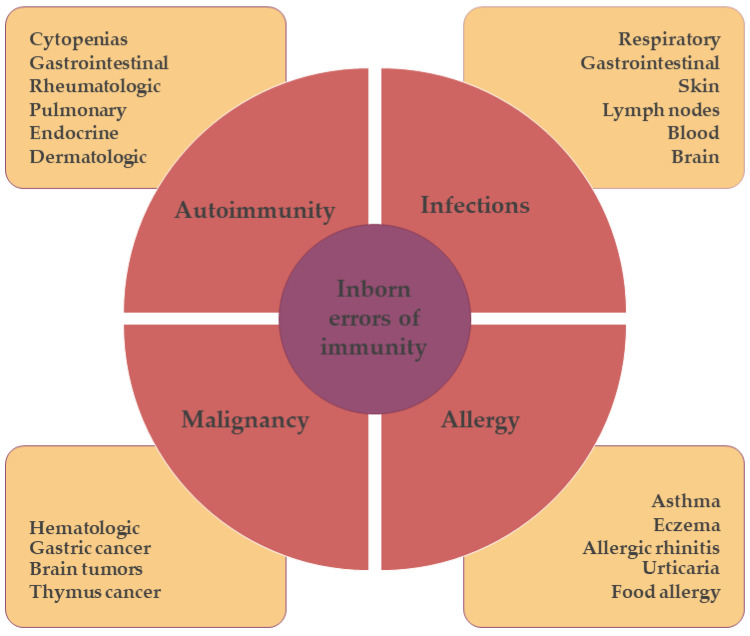
Schematic representation of the clinical course in patients with inborn errors of immunity. These patients may present with different clinical manifestations, such as severe infections (e.g., respiratory or gastrointestinal infections), neoplasms (e.g., hematologic malignancies—various types of leukemia, myeloma, and lymphoma; gastric cancer; brain tumors; thymic cancer; etc.), and/or autoimmune diseases (e.g., autoimmune thyroiditis or diabetes, eczema, autoimmune polyendocrinopathy, candidiasis and ectodermal dystrophy (APECED), immunodysregulated polyendocrinopathy enteropathy X-linked syndrome (IPEX)).

Elevated immunoglobulin E (IgE) is a hallmark of allergic diseases. However, high IgE levels are also found in a number of infectious diseases such as parasite infections, human immunodeficiency virus (HIV) infection, Mycobacterium tuberculosis, cytomegalovirus, Epstein–Barr virus, leprosy, and candidiasis. Inflammatory diseases such as eosinophilic granulomatosis with polyangiitis and Kawasaki disease are also characterized by elevated IgE levels. In addition, high IgE levels can be found in neoplasms such as Hodgkin’s lymphoma and IgE myeloma. Other diseases associated with elevated serum IgE levels include cystic fibrosis, nephrotic syndrome, bone marrow transplantation, graft-versus-host disease, and bullous pemphigoid. Tobacco smoking and the use of aztreonam or penicillin G may lead to an increase in total IgE levels [1]. Distinguishing between a child with atopic disease with recurrent infections and a child with an inborn error of immunity (IEI; also called primary immunodeficiency (PID)) can be quite difficult. It is important to note that allergic disease can be the clinical presentation of IEI. Inborn errors of immunity represent a growing group of diseases characterized by various combinations of severe and/or recurrent infections, inflammation, atopy, autoimmunity, lymphoproliferation, and malignancy. IEIs are predominantly monogenic disorders caused by mutations in genes responsible for immune defense and immunoregulation. In some IEIs, allergic symptoms may dominate the clinical picture (Figure 1) [2,3]. The allergic triad defined by elevated IgE, eosinophilia, and eczema is shared by several IEIs that may be misdiagnosed as common allergic diseases [4]. Depending on the predominant clinical and laboratory features, IEIs associated with atopic phenotypes, also known as primary atopic disorders, can generally be classified into several different phenotypes: (1) hyper-IgE syndromes (HIES); (2) Omenn syndrome (OS); (3) Wiskott–Aldrich syndrome (WAS) and WAS-like conditions; (4) immune dysregulation, polyendocrinopathy, enteropathy, and X-linked (IPEX) and IPEX-like conditions; (5) CBM-opathies—CADINS, CARD14 deficiency, and MALT1 deficiency; and (6) other IEIs presenting with allergic manifestations such as selective IgA deficiency, MyD88 deficiency, NEMO deficiency, and others [2,5].

In a recent study, allergic comorbidities were found in almost 40% of patients with antibody immunodeficiency and in 20% of patients with combined immunodeficiency [6]. We hypothesized that elevated IgE might be the reason for these allergic manifestations and therefore investigated IgE and IEI in our patients.

## 2. Materials and Methods

We performed a retrospective study that included 385 children (age range 3 months–17.9 years) referred to the clinic with suspected immunodeficiency. A 9-year period was covered—from January 2014 to December 2022. Most children were referred for evaluation because of recurrent respiratory infections. Interestingly, almost half of them had concomitant atopy, and other comorbidities included autoimmunity and malignancy. The following diagnostic tests were performed in each child—microbiological examination of throat and nasal swabs or sputum (if available), complete blood count, differential blood count, flow cytometric immunophenotyping with a wide range of monoclonal antibodies, and serum immunoglobulins and their subclasses—with an automated analyzer. Immunoglobulin levels were compared with age-adjusted normal reference ranges. In addition, antibody response to vaccines, evaluation of T-lymphocyte function, tests for autoantibodies, phagocyte activity, and genetic analysis for IEI were performed, as appropriate. Genetic analysis included whole-exome sequencing (Sanger method), examining all genes associated with a documented IEI. Immunological testing was performed when there was no evidence of current infection (laboratory—normal serum CRP and IL6 and no clinical signs of infection).

Statistical analysis of the data was performed using the Statistical Analysis Software Package (SPSS) version 26 (2021), IBM (Armonk, NY, USA) and Excel version 2013, Microsoft (Redmon, WA, USA). The analysis included descriptive statistics, tests to determine the normality of the distribution (Kolmogorov–Smirnov, Shapiro–Wilks), tests to compare quantitative indicators in different groups (comparison of means), and correlation analysis (χ-method, analysis of variance—ANOVA). The significance level chosen was α = 0.05, i.e., for values of *p* < α the null hypothesis is rejected.

## 3. Results

Of the total of 385 children, 214 (56%) were boys. The boys were slightly younger than the girls, with a mean age of 5.49 ± 3.85 years vs. 6.08 ± 3.84, respectively, but the difference was not statistically significant (*p* = 0.81). Overall, the mean age of the group was 5.75 ± 3.85 years. Regarding family history, we found that 33% were burdened with allergic diseases, 17% with autoimmune diseases, and 7% with malignant diseases. Clinical characteristics are shown in Table 1.

**Figure 2:**
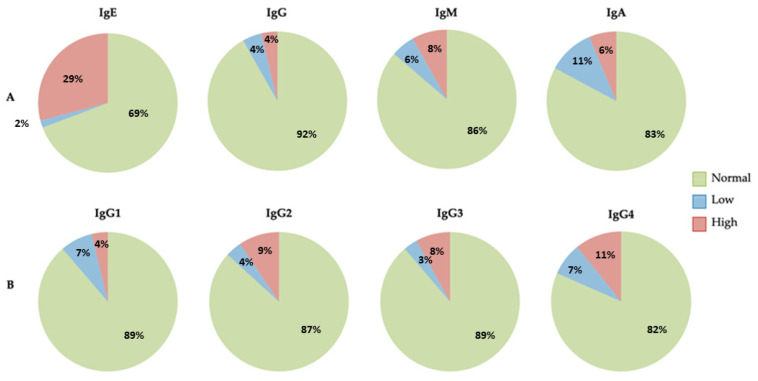
(**A**) The graph shows serum immunoglobulin IgE, IgG, IgM, and IgA levels. (**B**) The graph shows serum immunoglobulin IgG subclass levels.

We found no abnormal immunological parameters in 20 children (5.19%). Of the remaining 365 children, elevated and highly elevated IgE levels were found in 105 (28.78%), whereas IgE levels were extremely low (<2.5 kU/L) or absent in 8 children (2.19%). Low IgA levels were found in 40 children (10.95%), whereas they were elevated in only 21 (5.75%). Low IgM levels were detected in 22 children (6.02%), whereas elevated levels were found in 31 (8.49%). For IgG, elevated or decreased levels were found in 15 children (4.1%). As for IgG subclasses, decreased IgG1 was found in 26 children (7.12%) and increased in 16 (4.38%), decreased IgG2 in 15 patients (4.1%) and increased in 34 (9.38%), decreased IgG3 in 11 children (3.01%) and increased in 30 (8.21%), and decreased IgG4 in 25 patients (6.84%) and increased in 41 (11.23%) (Figure 2). The reference values used to label the patients with low, normal, or high immunoglobulins stratified by age are shown in Table 2.

We diagnosed primary immunodeficiency in 45 children (12.32%). Ten children were diagnosed with selective IgA deficiency (sIgAD), six with common variable immunodeficiency (CVID), three with X-linked agammaglobulinemia (XLA), three with hyper IgE syndrome (HIES), three with transient neutropenia (TrN), three with T-cell immunodeficiency (TCID), three children with selective IgG subclass deficiency, two with ataxia telangiectasia (AT), two with Di George syndrome, two with LAD deficiency, one with Netherton syndrome, one with STING -associated vasculopathy with onset in infancy (SAVI), one with C8 complement deficiency, one with DNA ligase IV (LIG4) syndrome, one with X-linked immunodeficiency with mutation in SD2S1A EBV–positive, one with Casteman disease (CD), one with CARD11 mutation, and one with FOXN1 immunodeficiency. Table 3 shows the number of patients with PID with low or high immunoglobulins.

**Figure 3:**
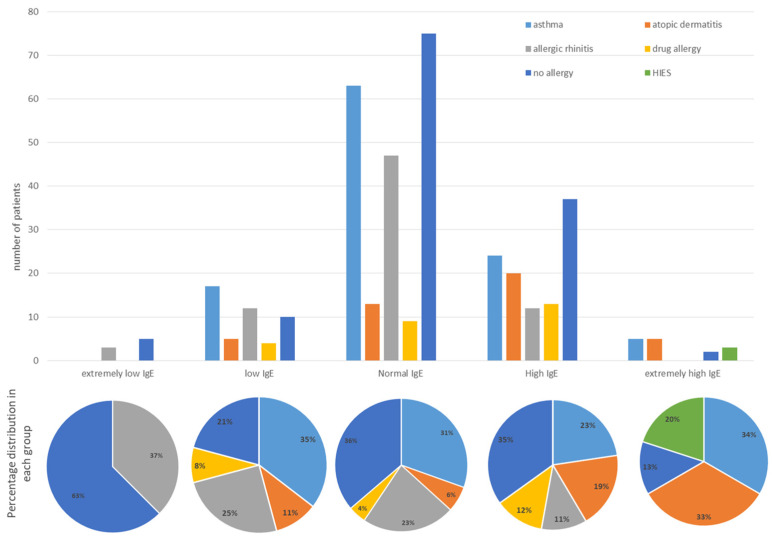
Number of patients with atopic comorbidities according to IgE group (HIES—hyper IgE syndrome) and percentage distribution of comorbidities in each group.

In addition, we focused on IgE levels in our study and divided patients into five groups: extremely low (below 5 IU/L) or absent IgE levels—8 children, low IgE levels—47 children (below 30 IU/L), normal IgE levels—205 children, elevated IgE levels—92 children, and extremely elevated IgE levels (above 1000 U/L)—13 children. The children’s atopic comorbidities were distributed differently depending on the group, and the results were statistically significant (*p* = 0.0062, Chi-square). As expected, only the group with extremely elevated IgE had patients with HIES, and we did not look for statistical differences in this comorbidity for the different groups but focused on differences in the frequency of patients with asthma, atopic dermatitis, allergic rhinitis, drug allergies, or no allergies according to IgE levels (Figure 3). Patients with drug allergy and atopic dermatitis were more likely to have elevated IgE levels, whereas patients with allergic rhinitis were more likely to be in the normal or low IgE groups on a percentage basis. Patients with bronchial asthma were equally likely to fall into the normal or elevated IgE groups, with nearly a quarter having asthma symptoms. However, the elevated IgE group had the most patients with more than one atopy—21 children (21.73%) versus 2 patients in the normal IgE group (0.9%) and 1 in the low IgE group (2.08%).

When we evaluated the IgE levels of patients with confirmed PIDs, we found elevated IgE levels in 10 children (22.22%) and the same number of children (10–22.22%) with low IgE, although 3 of them had extremely low IgE levels (6.66%), whereas the rest had normal IgE levels. Of the patients with extremely low IgE, one had CVID, one had AT, and one had selective IgG2 deficiency. The other seven children with low IgE included two with CVID, two with sIgAD, two with XLA, and one with TrN. In the group with elevated IgE, three children with HIES had extremely high IgE, and the others with high IgE included children with sIgAD, two children with TCID, the child with Netherton syndrome, the child with CARD11 mutations, and the child with SAVI. The small number of groups did not allow us to generate in-depth statistics. But, as in other reports, our CVID patients were more likely to be in the low IgE group, whereas those with TCID were more likely to have higher IgE. However, there were no statistically significant correlations between IgE levels and levels of other immunoglobulins.

Of the 13 patients with extremely high IgE levels—above 1000 U/mL—we performed genetic testing, and only 3 had a confirmed HIES. Of the other 10, 5 patients were diagnosed with bronchial asthma and 5 with atopic dermatitis. The percentage of atopic comorbidities in this group was also high, but this came from the small numbers in this group, where two patients had a percentage of 15.38%.

In the present study, we measured IgE, IgG, IgA, IgM, and IgG subclasses in blood samples from 385 children with suspected IEI. We found that almost one-third of our patients had elevated serum IgE levels. Most of the cases with elevated IgE were children with underlying atopy—mainly atopic dermatitis and, to a lesser extent, bronchial asthma—but 40.12% (37 children) had no atopy at all. At the same time, we found patients with allergic diseases and normal IgE levels, although it should be noted that our cohort of children was relatively young, with an average age of 5.75 years. Patients with IEI may have elevated IgE levels not only in HIES. Our results demonstrate once again that allergic phenotype should not be underestimated and that children with more severe allergic diseases should be evaluated for an underlying inborn error of immunity.

## 4. Discussion

Patients with IEI with atopic phenotype present with specific clinical manifestations and laboratory findings that must be carefully analyzed to identify the correct disorder. It is important to evaluate the presence or absence of a positive family history for primary immunodeficiencies and/or consanguinity, as well as the presence of prenatal and perinatal factors such as infections, smoking, e-cigarette use, alcohol, and drugs during pregnancy as risk factors for adverse birth outcomes.

**Figure 4:**
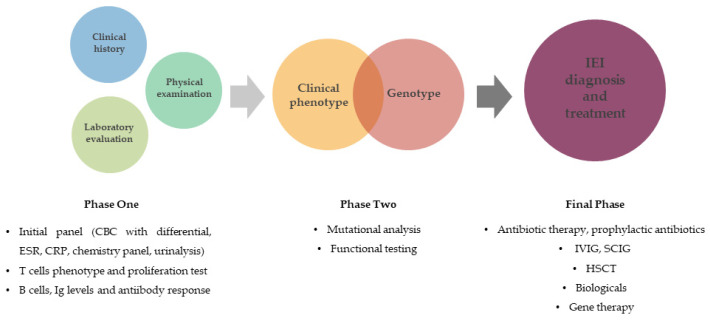
Diagnostic approach to inborn errors of immunity. This diagnostic process must be fully performed in order to make the correct diagnosis and initiate optimal treatment. CBC—complete blood count, CRP—C-reactive protein, ESR—erythrocyte sedimentation rate, HSCT—hematopoietic stem cell transplantation, IEI—inborn error of immunity, IVIG—intravenous immunoglobulin, SCIG—subcutaneous immunoglobulin.

Many IEI disorders are inherited in an autosomal recessive manner. Therefore, individuals with autosomal recessive disorders and their families should be told that marriages between relatives increase the likelihood that children will be affected. There are several features that raise suspicion of immunodeficiency and suggest referral to a specialist for investigation. A detailed history, a thorough physical examination, and laboratory tests to assess immune system function are the first steps toward a correct diagnosis (Figure 4) [7].

Immunodeficiencies associated with atopy include selective immunoglobulin A deficiency (sIgAD), common variable immunodeficiency (CVID), chronic granulomatous disease (CGD), and DiGeorge syndrome, as well as several other less common forms [8]. As previously published, a child with severe atopic dermatitis and IEI should be screened for HIES, Wisscott–Aldrich syndrome, Omen syndrome, IPEX syndrome, Netherton disease, and DiGeorge syndrome [9]. Our two patients with DiGeorge syndrome did not have atopic dermatitis, and although their CD3 levels were below 50% of age-adjusted levels, they also had normal IgE levels [10]. We might suggest that atopy in DiGeorge syndrome is more complicated than has been reported. Extremely elevated IgE levels and skin involvement always prompt us to look for HIES, and we refer patients for STAT3 mutation analysis [11,12]. However, the child with the most severe atopic dermatitis in our group, who also had extremely elevated IgE levels, was not an HIES patient, but CARD11 mutations were confirmed. This mutation is relatively new for patients with severe dermatitis and should also be part of the study panel, as it would allow for a different treatment approach [13]. In addition to CARD11, STAT3 and other transcription factor defects such as FOXP3 should also be sought [14,15]. We found that of our patients with skin involvement, similar to atopic dermatitis, all were children with IEI and elevated IgE, but two were children with sIgAD. Patients with sIgAD are more likely to suffer from asthma, upper respiratory allergies, and food allergies [16,17]. These findings were also confirmed in the 10 patients with sIgAD. Eight of them were evaluated for recurrent respiratory infections, and half of them were confirmed to have asthma, three patients suffered from drug allergy and two from allergic rhinitis, and four patients had more than one atopic comorbidity. Two patients with sIgAD had low IgE and were the only ones without atopic comorbidity, whereas the rest had normal (5 patients) or high (3 patients) IgE levels.

The presence of atopic disease (asthma, eczema, allergic rhinitis, and urticaria) may be a feature of CVID in children compared with CVID in adults. Absence of immunoglobulin E (IgE) is common in both children and adults, with 97% failing to detect allergen-specific IgE [18]. In the two pediatric series, 38% of patients in one of the cohorts had signs of allergic disease, including asthma, food allergy, eczema, urticaria, and rhinitis, and 83% in the other cohort had asthma [19]. In a series of 45 patients, 5 had atopic dermatitis and 6 had eosinophilic esophagitis [20]. All of our children with CVID were referred to the clinic for recurrent respiratory symptoms, but only one had asthma and food allergy and normal IgE levels. In a recent Iranian study, patients with CVID were found to have mainly food allergy or atopic dermatitis rather than bronchial asthma despite recurrent respiratory symptoms [21]. Skin involvement in CVID is gaining attention, and some authors state that it is not that rare [22], but none of our CVID patients had any skin problems. Similar to other studies, half of them had very low IgE levels, whereas the rest had normal IgE levels.

It should be noted that, in our group, a relatively high percentage of patients with allergic rhinitis and asthma was found in the low or normal IgE group. There are several possible explanations for this, one of which is the age of our patients, with the average age being almost 6 years, and the fact that there were patients with viral-related asthma, which is not necessarily related to IgE [23]. On the other hand, there are publications indicating that serum IgE is low in the initial stage of allergic rhinitis, when inflammation is still on a local basis [24].

Studies have found an association between low serum IgE levels and levels of one or more immunoglobulin classes (i.e., IgG, IgA, and/or IgM) below the age-appropriate reference range. Consistent with these reports, a recent literature review concluded that low IgE levels should be considered a clinical laboratory “flag” for screening for antibody deficiency [25]. The review shows that low IgE type 1 was associated with antibody deficiency (low IgG and IgA) and susceptibility to infection and/or autoimmunity. When a secondary cause for the antibody deficiency was unlikely, these patients were often diagnosed with CVID. The only IgG subclass that was not significantly altered in low IgE type 1 was IgG3. Low IgE type 2 is characterized by particularly low IgG3 and IgG4 levels but shows no decrease in other IgG subclasses. Total IgG and IgA levels were normal in patients with low IgE type 2. The patients with low IgE type 2 were referred for allergy testing. A review of the records revealed that the main symptoms were rhinitis and asthma [25]. We did not find a correlation between high IgE levels and other immunoglobulins, but when IgE levels were low and IEI was confirmed, these children also had low IgG, IgM, and IgA levels (Table 3).

An elevated IgE level (100 ≥ IgE < 1000 kU/L) indicates allergy, eczema, or chronic skin infection. A very elevated IgE level (1000 ≥ IgE < 10,000 kU/L) is not uncommon in patients with active eczema but is suggestive of a monogenic atopy disorder or one of the hyper-IgE immunodeficiency syndromes in which extremely high total serum IgE levels (IgE ≥ 10,000 kU/L) may be found. A very high IgE level (e.g., >2000 international units/mL) in a patient with recurrent bacterial or fungal infections and dermatitis would raise suspicion of a hyper-IgE syndrome and several other IEI-like signal transducer and activator of transcription 3 (STAT3), interleukin 21 receptor (IL21R), interleukin 6 receptor (IL6R), and interleukin 6 signal transducer (IL6ST) deficiencies; caspase recruitment domain family member 11 (CARD11) dominant negative defect; phosphoglucomutase 3 (PGM3) deficiency; Wiskott–Aldrich syndrome; Omenn syndrome; IPEX (immune dysregulation, polyendocrinopathy, enteropathy, X-linked) syndrome; dedicator of cytokinesis 8 (DOCK8) deficiency; lipopolysaccharide-responsive-beige-like anchor (LRBA) deficiency; and a gain-of-function phenotype associated with enhanced phosphorylation and transcriptional activity of STAT6 (signal transducer and activator of transcription 6) [26].

IgG subclasses are not routinely studied, although IgG subclass deficiency is a common finding among patient populations with recurrent sinopulmonary infections [27]. IgG subclass deficiencies are most strongly associated with IgA deficiency. About 15% of patients with IgA deficiency also have a deficiency of IgG subclasses, such as IgA, IgG2, and IgG4 deficiency [28]. Associations have also been reported between IgG subclass deficiency and ataxia–telangiectasia and selective IgM deficiency. IgG subclass deficiencies are also found with a high frequency among patients with chronic airway diseases, particularly asthma and chronic obstructive pulmonary disease [29]. Atopic diseases and autoimmune conditions, such as vasculitis and cytopenias, are more common in patients with IgG subclass deficiency.

Recent data suggest that IgG4 may be an important biomarker in cancer. IgG4 is the least abundant subclass of IgG in normal human serum but can reach elevated levels during chronic antigen exposure or inflammation. IgG4 are widely considered to be anti-inflammatory antibodies that play a positive and protective role in allergies and helminth infections. In allergies, IgG4 are responsible for neutralizing allergens and inhibiting IgE signaling and are associated with immune tolerance to allergens in allergen immunotherapy (AIT).

On the other hand, in IgG4-related diseases such as sclerosing pancreatitis and autoimmune pancreatitis, a pathogenic role of IgG4 cannot yet be completely excluded. In contrast, the anti-inflammatory properties of IgG4 could have a negative impact in the context of cancer. Because tumors are characterized by chronic inflammation with prolonged exposure to tumor-associated antigens, elevated IgG4 has been demonstrated in extrahepatic cholangiocarcinomas, pancreatic cancers, melanomas, and glioblastomas. These tumor types are characterized by Th2-loaded expression of mediators known to stimulate B cells to produce IgG4. Tumor-specific IgG4 can act as a blocking antibody that competes with inflammatory antibodies such as IgE or IgG1 to bind to antigen or in the form of immune complexes that target the inhibitory receptor FcγRIIb to dampen inflammation. IgG4 antibodies in cancer may thus have a downside compared with their association with tolerance induction to allergens [30]. Elevated bloodstream IgG4 levels in patients are associated with risk of disease progression in melanoma [31]. The percentage of survivors decreases with higher IgG4 levels. Therefore, it could probably be used as a biomarker in cancer patients and for follow-up in the search for metastases. These findings raise the question of whether allergen-specific immunotherapy, which decreases specific and total IgE and increases IgG4, promotes the development of cancer. A history of malignancy is a contraindication for AIT, but a family history of cancer should probably be considered and discussed with patients to make a decision about treatment.

According to epidemiological studies, atopic and allergic patients are less likely to develop certain types of cancer, such as lymphoma, neuroblastoma, pancreatic cancer, and melanoma [32]. Mouse models show that higher IgE levels are critical for the survival of mice with cancer [33]. IgE effector cells are cytotoxic against tumors [34]. On the other hand, ultra-low IgE is a risk for cancer. For normal total serum IgE levels (kU/L) of 2.5 ≥ IgE < 100, IgE deficiency is assumed if IgE < 2.5 kU/L. In a retrospective study, the authors showed that CVID and IgE-deficient patients are more likely to develop lymphoma and uterine cancer, whereas non-CVID + IgE-deficient patients are more prone to malignancies—lymphoma, myeloma, breast cancer, and kidney cancer [35]. All children included in our study with neoplasms as concomitant disease had normal IgE levels, but this may have been due to the absence of patients with IgE myeloma, Hodgkin’s and non-Hodgkin’s lymphoma, or cancers described in patients with elevated IgE and tumors [36,37].

The overall risk of developing cancer in patients with IEI is less than twice that in the general population and is estimated to be between 4 and 25% [38]. The incidence of malignancy in CVID patients is estimated to be about 10%, with values ranging from 1.5 to 20.7% in various studies [39]. The Australasian Society of Clinical Immunology and Allergy (ASCIA) study found a 7-fold increased risk of stomach cancer, a 12-fold increased risk of non-Hodgkin’s lymphoma (NHL), and a 146-fold increased risk of thymic cancer [40]. The mechanisms of increased susceptibility to NHL and thymic cancer are not clear. Impaired immunity to herpes viruses, chronic inflammation, and DNA repair defects are probably known predisposing factors in this patient group [41]. Altered function of the P53 protein produced by the tumor suppressor gene TP53 is associated with gastric malignancies in patients with CVID. Other factors, such as chronic Helicobacter pylori infection, pernicious anemia, achlorhydria, and decreased gastric IgA secretion, may also predispose these patients to the development of gastric malignancies.

Patients with IEI are generally more likely to have cancer that has spread or is widespread at the time of diagnosis and therefore have a poorer prognosis. Patients with certain conditions, such as ataxia telangiectasia, Nijmegen breakage syndrome, and generalized variable immunodeficiency, should be informed of their radiosensitivity, which may increase the risk of developing malignancy. These patients should consult their immunologist before agreeing to any diagnostic or therapeutic intervention involving radiation suggested by other clinicians. Clinicians should weigh the risks and benefits of the procedure in the context of the underlying disease, the need for the procedure, replacement with nonradiation techniques (e.g., sonography and magnetic resonance imaging), and, when possible, in vitro analysis of the patient’s radiation sensitivity [42].

## 5. Conclusions

Advanced immunologic testing requires expertise to perform and interpret, is not widely available, and is often costly. Therefore, immunologic testing is best performed in a stepwise fashion, and referral to an immunologist should be made as early as possible. When the specific laboratory tests performed are inconclusive or it is difficult to classify the clinical phenotype and a definitive diagnosis cannot be made, genetic testing may be helpful. Gene panels, whole-exome sequencing, or whole-genome sequencing can be performed. Based on the genetic studies, overlaps were found between known atopy-related genes as described in the Human Gene Mutation Database and disease-causing genes of monogenic PIDs [43]. Identification of genetic mechanisms playing a role in the pathogenesis could open up the knowledge for novel therapeutic targets and a more tailored therapy of these diseases.

IgE is known as a marker of allergy or parasitic infection, but based on previously published data and this study, we believe it is time to change the clinical approach and, in addition, screen these children for IEI. In cases of extremely high IgE levels, genetic testing is mandatory to confirm or exclude some of the HIES.

IEI could be misdiagnosed based on the predominant clinical features of atopy. Without consideration of underlying IEI, some individuals remain undiagnosed, resulting in a high risk of morbidity and mortality. An underlying IEI with atopic phenotype should be considered, especially in severe cases of atopic disease with concurrent signs of autoimmunity and recurrent infections, unusual clinical course, and lack of response to classic treatment strategies. When IEI with atopic phenotype is suspected, a comprehensive immunologic evaluation is required, and genetic testing is essential to identifying the specific genetic abnormality. Good collaboration between pediatricians, allergists, and immunologists is necessary to correctly diagnose IEI and initiate optimal targeted therapy.

Limitations of the study: There are fewer patients with particularly low and high IgE levels. Therefore, the correlation between atopic comorbidities and IgE levels may not reach statistical significance in this group.

## Figures and Tables

**Table 1:** Clinical characteristics and demography of the children in the study.

	Patient Results
Mean age (±SD), years	5.75 ± 3.847
Sex (m:f)	214:171
Leading reasons for suspected IEI and number of patients referred for it	Recurrent respiratory infections—296 Chronic diarrhea and GIT infections—21 Recurrent fever of unknown origin—15 Recurrent skin infections—10 Unexplained anemia—4 Lymphoproliferative disease (unusual presentation or type for the age)—22 Immune thrombocytopenia—14 Tumor—3
Comorbidities	No comorbidities—80 Allergic diseases (incl. asthma)—252 Congenital heart disease—6 Epilepsy—4 Diabetes—3 Anemia—4 Thrombocytopenia—14 Neoplasm—22
Allergic diseases and HIES	Asthma—109 Allergic rhinitis—74 Atopic dermatitis—43 Drug allergy—26 HIES—3 No allergy—130
Family history for atopy	No family history—106 Atopy—83 Autoimmunity—33 Autoimmunity and atopy—27 Neoplasm—9 Autoimmunity and neoplasm—1 Atopy and neoplasm—13 Autoimmunity and atopy and neoplasm—4 Bronchial asthma—62 Other chronic diseases (diabetes, arterial hypertension, epilepsy, etc.)—47

**Table 2:** Reference values for immunoglobulins stratified by age.

Turbimetry	0–6 Months	6 Months–2 Years	2–4 Years	4–6 Years	6–10 Years	10–12 Years	12–14 Years	Over 14 Years
IgA g/L	0.2–1.0	0.2–1.0	0.2–1.0	0.3–1.95	0.5–2.5	0.5–2.0	0.6–3.6	0.6–3.5
IgG g/L	3.2–12.7	3.2–12.7	4.7–12.5	5.3–13.4	5.4–16.1	5.5–14.9	6.6–14.9	5.5–14.4
IgM g/L	0.2–1.0	0.4–2.2	0.3–2.0	0.3–2.0	0.5–1.8	0.5–1.8	0.35–2.42	0.35–2.42
IgE IU/mL	<60	<60	<60	<100	<100	<100	<120	<120
IgG1 g/L		1.3–6.33	3.1–9.4	3.1–9.4	2.88–9.18	4.3–10.6	3.4–11.5	3.15–8.55
IgG2 g/L		0.3–1.29	0.3–2.2	0.6–3.4	0.44–3.75	0.4–3.5	1.0–4.5	0.64–4.95
IgG3 g/L		0.1–0.6	0.1–0.6	0.1–1.2	0.15–1.5	0.1–1.7	0.3–1.2	0.23–1.96
IgG4 g/L		0.01–0.2	0.01–0.5	0.02–1.1	0.01–1.1	0.01–1.1	0.04–1.3	0.11–1.57

**Table 3:** Number of patients with confirmed IEI distributed according low or high levels of different immunoglobulins.

Immunoglobulins	IgA	IgM	IgG	IgE
Low	sIgAD—10 CVID—5 XLA—3 TrN—2 AT—2 CD—1	CVID—6 XLA—3 CD—1 TrN—1 TCID—1	CVID—6 XLA—3 Di George—1 TrN—1 TCID—1 CD—1	CVID—3 XLA—2 sIgAD—2 TrN—1 AT—1 IgG2 deficiency—1
High	LAD deficiency—2 SAVI—1	AT—2 LAD deficiency—2 SAVI—1 sIgAD—1 HIES—1	LAD deficiency—2 sIgAD—2 SAVI—1 HIES—1 TCID—1	HIES—3 sIgAD—2 TCID—2 SAVI—1 Netherton—1 CARD11—1

## Data Availability

The data presented in this study are available on request from the corresponding author. The data are not publicly available due to restrictions, e.g., privacy or ethics.
